# Multi-Response Optimization of Electrical Discharge Machining Using the Desirability Function †

**DOI:** 10.3390/mi10010072

**Published:** 2019-01-20

**Authors:** Rafał Świercz, Dorota Oniszczuk-Świercz, Tomasz Chmielewski

**Affiliations:** Institute of Manufacturing Technology, Warsaw University of Technology, 00-661 Warsaw, Poland; doo@meil.pw.edu.pl (D.O.-Ś.); t.chmielewski@wip.pw.edu.pl (T.C.);

**Keywords:** electrical discharge machining, electrical discharge machining (EDM), surface roughness, surface integrity, optimization, desirability function

## Abstract

Electrical discharge machining (EDM) is a modern technology that is widely used in the production of difficult to cut conductive materials. The basic problem of EDM is the stochastic nature of electrical discharges. The optimal selection of machining parameters to achieve micron surface roughness and the recast layer with the maximal possible value of the material removal rate (MRR) is quite challenging. In this paper, we performed an analytical and experimental investigation of the influence of the EDM parameters: Surface integrity and MRR. Response surface methodology (RSM) was used to build empirical models on the influence of the discharge current *I,* pulse time *t*_on,_ and the time interval *t*_off_, on the surface roughness (*Sa*), the thickness of the white layer (WL), and the MRR, during the machining of tool steel 55NiCrMoV7. The surface and subsurface integrity were evaluated using an optical microscope and a scanning profilometer. Analysis of variance (ANOVA) was used to establish the statistical significance parameters. The calculated contribution indicated that the discharge current had the most influence (over the 50%) on the *Sa,* WL, and MRR, followed by the discharge time. The multi-response optimization was carried out using the desirability function for the three cases of EDM: Finishing, semi-finishing, and roughing. The confirmation test showed that maximal errors between the predicted and the obtained values did not exceed 6%.

## 1. Introduction

Electrical discharge machining is a precision method of manufacturing hard, complex shaped, conductive materials. The removal mechanism of the material in EDM is the result of the electrical discharge, which causes melting and evaporation in the local surface layers of both the workpiece and the working electrode. Owing to the impact of the thermal and chemical processes, the electrical discharge properties of the surface layer of the material are changed [1,2,3]. Craters form a specific surface texture. Surface roughness parameters directly depend on the discharge current and the time pulse [4]. Heat flux changes the surface integrity. Metallographic images of the machined samples of tool steel show new layers, i.e., the external melting layer (white layer), the heat-affected zones, and the tempered layer. The white layer is non-homogeneous and discontinuous and may vary in thickness on the analyzed sample. Discontinuity of the melted layer is caused by the random occurrence of electrical discharge and the machining conditions. This layer is characterized by high variations in thickness and microstructure defects of the material, such as micro-cracks. Microcracks are an undesirable effect, resulting in reduced fatigue resistance and corrosion resistance. It is important to choose the appropriate parameters and processing conditions to obtain the smallest thickness of the white layer or its complete elimination. The quality of the surface after the EDM process does not always meet expectations [5,6]. Therefore, additional technological operations are used to change the surface integrity. Some of the most applicable operations are electrochemical machining [7,8], laser surface modification [9,10], applying coatings [11,12], or the use of hybrid machining [13,14] or non-conventional finishing [15,16,17,18]. However, the use of additional technological operations significantly increases the production costs. Therefore, this work analyzed the impact of the following parameters of the EDM process: discharge current, pulse time, and pulse interval. Furthermore, the optimization of EDM using the desirability function will allow for the selection of the most favorable processing conditions, which reduce the use of additive treatments to the bare minimum.

Electrical discharge machining belongs to a group of non-conventional manufacturing techniques. The material is removed from the workpiece using electrical discharges occurring between the working electrode and the workpiece. Owing to the conducted thermal energy from the electrical discharge, the local temperature increases (in the range of 8000–12,000 °C), leading to the melting and evaporation of a small volume of the surface workpiece and the working electrode. Then, a collapsing plasma channel at the end of the discharge induces high-pressure waves that rinse the molten and evaporated metal [19]. The physics of the material removal phenomenon in EDM is complex. A model of the EDM process proposed by Izquierdo et al. [20], shows that by using the superposition of multiple discharges and calculating the temperature fields inside the workpiece, it is possible to predict the surface roughness parameters and the material removal rate. One of the main problems in modeling is the appropriate determination of the influence of EDM parameters like discharge voltage, discharge current, and pulse time on ionization and growth of the plasma channel. Information about the percentage of discharge energy devoted to the heat flux, the mechanism of plasma channel growth, and the temperature which facilitates material removal, is used to model not only surface roughness, but also to build models of structural changes in the surface layers and their thickness [21]. Ming et al. [22] analyzed the distributions of the energy of the workpiece for different materials like Al 6061, Inconel 718, and SKD11. The authors indicated that the analyses of energy efficiency simultaneously with MRR, could be used in existing thermal–physical models to improve the technical performance of the models. Theoretical models of EDM are significant because the models analyze the processes and their implication on the manufactured material. However, the application of the theoretical model in the manufacturing process is not always possible. Given the complexity in describing the physical phenomena of EDM, such as the random distribution of the electric field and the temperature field, the formation of the plasma channel, and changes in the properties of the dielectric, mathematical models have been built based on the empirical studies. Several process variables promote the application of the optimization method to achieve high productivity.

Optimization of the EDM machining process can be carried out using various methods like response surface methodology [23,24], Taguchi analyses [25], artificial networks [26], grey-based response surface methodology [27,28], and the Deringer desirable [29] or hybrid methods [30,31]. One of the most common manufacturing processes is the response surface methodology (RSM). RSM is extensively used in an analytical and industrial application like turning [32,33], milling [34], welding [35], grinding [36], and erosion machining [37]. Ghodsiyeh et al. [38] used RSM to optimize the wire electrical discharge machining of the titanium alloy Ti-6Al-4 V. Presented results indicated that the pulse time had the highest impact on surface roughness, whilst discharge current had the same role for the wire wear ratio and white layer thickness. Alavi et al. [39] analyzed the influence of the EDM parameters on tool wear, crater size, and microhardness on the titanium alloy Ti-6Al-4V. The presented research indicates that the main effect on the crater size was the discharge voltage, whilst the capacitance was the most important for tool wear and surface micro-hardness. Increasing the capacitance caused a reduction in tool wear and an increase in the micro-hardness of the machined surface. Selvarajan et al. [40] investigated the possibilities of the EDM composite Si_3_N_4_-TiN. The authors used RSM to determine the optimal parameters for the machining of ceramic composites. The conducted research showed that the MRR and surface roughness of the manufactured parts depended on the value of the discharge current and the pulse time, and the results were consistent with the results for the processing of tool steel [41]. Kumaran et al. [42] used a grey fuzzy logic approach to optimize the EDM parameters during the machining of carbon fiber reinforced plastic composite. Their research showed that established optimal parameters in ultrasonic-assisted EDM allowed improvements in the deburring rate, with a simultaneous improvement in the tool wear rate (TWR). Gu et al. [43] indicated that the machining of new alloys, which have a high melting point and good thermal conductivity, like titanium–zirconium–molybdenum, require optimizations of the EDM in connection with the analysis. The presented results showed that the crater diameter was much smaller than the plasma-affected zone. To improve the machining performance (*Ra,* MRR), the response surface methodology was used. Dang [44] proposed the Kriging regression model and particle swarm in the optimization of EDM of P20 steel. The authors indicated that the Kriging model could capture the nonlinear characteristics and was better able to obtain the optimum parameters for the MRR, tool wear, and surface roughness. Mohanty et al. [45] pointed out that the choice of the electrode material should be considered in the optimization of the EDM process. The authors found that in the machining of Inconel 718, the material removal rate and tool wear could be improved by using a graphite electrode and to improve surface integrity, the better choice was a brass and copper electrode. Using the utility concept and the quantum particle swarm optimization (QPSO) algorithm, the optimal parametric setting was developed with the objectives to maximize the MRR and minimize tool wear, surface roughness, and radial overcut. Research presented by Maity et al. [46] on the influence of EDM parameters on the thickness of the recast layer, material removal rate, and overcut on the machining of Inconel 718, showed that the optimization parameters using the RSM and Artificial Bee Colony algorithm gave an average prediction accuracy of about 3.5% in relation to confirmation tests. The predictive efficiency of neural networks may be affected by different factors like noise corruption, spatial distribution, and the size of the data used to construct the artificial neural network (ANN) model. Tripathy et al. [47], in order to optimize the powder mixed (SiC) electro-discharge machining of H-11 die steel, which seeks to maximize the MRR and minimize electrode wear and surface roughness, used a different method of optimization. The authors used the grey relational analysis and the technique for the order of preference by similarity, where the Technique for Order of Preference by Similarity to Ideal Solution (TOPSIS) solution achieved a similar effect of an improved performance of the quality characteristics. Nguyen et al. [48] investigated the influence of powder mixed (Ti) electro-discharge machining of SKD61, SKD11, SKT4 steel on the surface roughness (SR), MRR, and microhardness. The presented results showed that the addition of Ti powder in dielectric resulted in reduced SR and increased microhardness. The authors indicated that optimization with the Taguchi–TOPSIS made it difficult to select the optimal parameters. The presented research showed that the measured distance could lead to confusion in selecting the best alternative. A fuzzy analytic hierarchy process (AHP) and fuzzy TOPSIS method were used by Roy et al. [49] to optimize multiple responses of the material removal rate, tool wear, and tool overcut in EDM based on various process parameters. Kandpal et al. [50] investigated the influence of EDM parameters on the MRR, tool wear, and overcut of aluminum matrix composites. The authors showed optimization with the utility concept, which provided the collective optimization of both responses for improving the mean of the process. D’Urso et al. [51] proposed the optimization of EDM micro drilling using a cost index which combined two opposite effects of the material removal rate and tool wear. The minimization of the cost index enabled optimal working conditions. Parsana et al. [52] indicated that in the optimization process of EDM drilling, an important point to consider was the roundness of the holes. Using the RSM and passing vehicle search algorithm, normalized weights proved to be useful in obtaining the Pareto fronts for a combination of different objectives at a time. Research carried out by Hadad et al. [53] showed that the analysis of the optimal parameters of EDM machining, in addition to the electrical parameters, should also include the initial roughness of the working electrodes, which has significant effects on the machining performance during the finishing, semi-rough, and rough EDM processes. 

The published literature indicates that few studies have reported on the optimization of the EDM, which considers the surface 3D roughness parameter, white layer thickness, and the MRR in the three stages of machining: roughing, semi-finishing, and finishing, with the desirable function. Therefore, in this paper, a multi-response optimization of the EDM of tool steel 55NiCrMoV7 was conducted. This material has a wide range of industry applications on die matrices, matrix inserts, and hydraulic and mechanical press dies. The surface roughness was investigated using the 3D roughness parameter. The parameter, *Sa,* gives more information about the surface properties. In electrical discharge machining, the surface roughness is obtained by overlapping the craters of individual discharges. EDM with parameters corresponding to the roughing and semi-finishing operations results in surfaces which have different profiles on the cross-sections of samples. The calculated *Ra* parameters on the profile cross-sections may provide inaccurate results. 

The optimization of parameters was performed using the desirable function. Optimization of electrical discharge machining was divided into three cases: finishing, semi-finishing, and roughing. In each case, different goals were set. For finishing, the goal was to minimize the surface roughness (*Sa*) simultaneously whilst minimizing the thickness of the white layer (WL), with a possible maximized MRR. In the case of semi-finishing, the EDM goal was to obtain a specific value of the MRR whilst possibly minimizing the roughness (*Sa*), simultaneously minimizing the thickness of the white layer (WL). In the last case of roughing, the goal was to maximize the material removal rate with the possibility of minimizing the roughness (*Sa*) and the thickness of the white layer (WL).

The topic of the article focuses on describing the changes occurring in the material as a result of the local thermal processes due to electric discharges. Experimental studies allowed for a better understanding of the relationship between the changes in surface integrity of tool steel 55NiCrMoV7 and how to optimize the process to achieve a micron roughness and recast layer, with the possibility of achieving the maximal value of the material removal rate.

## 2. Materials and Methods 

Industry applications of electrical discharge machining are limited by the obtained specific surface integrity and low material removal rate. The purpose of the research was to develop the multi-response optimization of the EDM process of the tool steel 55NiCrMoV7 for three cases: finishing, semi-finishing, and roughing. Tool steel 55NiCrMoV7 was chosen because of its wide industry applications on die matrices, matrix inserts, and hydraulic and mechanical press dies. This material is characterized by high dimensional stability and crack resistance, with dynamically changing pressures and rapid heating and cooling during operation. Heat-treated samples of the tool steel (55 HRC) had the dimensions of 12 mm × 12 mm × 3 mm. Experimental studies were conducted on the electrical discharge machine, Charmilles Form 2LC ZNC (GF Solutions, Geneva, Switzerland). The electrode used was graphite (EDM-3 POCO), and the EDM fluid 108 MP-SE 60 was used as the dielectric. The present paper was focused on the selection of optimal parameters for EDM, which led to minimum surface roughness, as well as the thickness of the white layer and maximum productivity.

**Figure 1:**
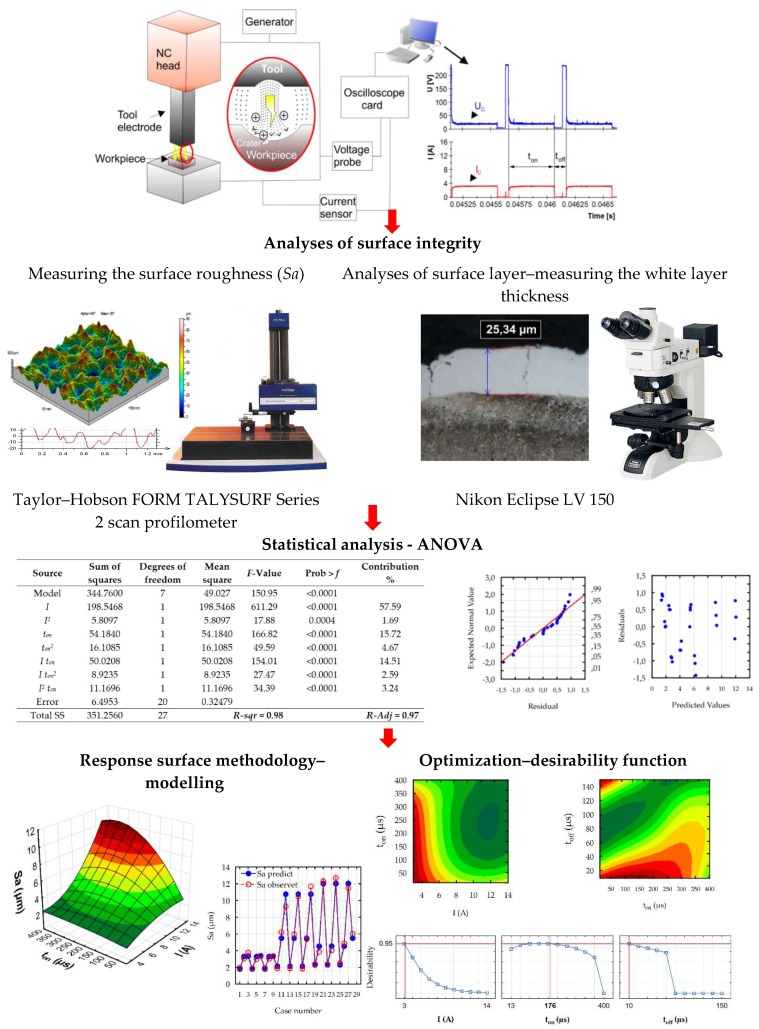
The schematic diagram of the experimental set-up.

The main object of the study was the optimization of the EDM process using statistical models on the influence of EDM parameters on surface roughness (*Sa*), the thickness of the white layer, and the material removal rate. To achieve this goal, experimental research was carried out using a completely orthogonal design of the experiment, three-level three parameters full factorial design. Choice of this type of experiment design allowed the reduction in the number of experimental runs required to generate sufficient information for a statistically adequate result. A schematic diagram of the experimental set-up is shown in Figure 1. Investigation of the surface roughness parameters after the EDM was carried out on a Taylor–Hobson FORM TALYSURF Series 2 scan profilometer (Taylor Hobson, Leicester, United Kingdom). The roughness parameter (*Sa*), the arithmetic mean of the deviations from the mean, was measured on a surface area of 2 mm × 4 mm with a discretization step (10 μm) in the *X*-axis and *Y*-axis. The *Sa* (average value of the absolute heights over the entire surface) parameter responded to the 2D roughness profile parameters *Ra*. This may be obtained by adding the individual height values, without regard to sign, and dividing the sum by the number of the data matrix, where *M* is the number of points per profile, *N* is the number of profiles, and *z*, *x*, *y* are the heights of the profile at a specific point.

(1) $$Sa=\frac{1}{NM}\overset{N=1}{\underset{x=0}{\sum }}\overset{M=1}{\underset{y=0}{\sum }}\left|z_{x,y}\right|$$

Metallographic surface structure studies were performed using a Nikon Eclipse LV 150 optical microscope (Nikon, Tokyo, Japan), coupled to an NIS-Elements BR 3.0 image analyzer (Nikon). Specimens were included in the resin, and were then machined with grinding and polishing. Micro-etching was performed with nital (5%) to reveal the microstructure of the material. The maximum thickness of the white layer in sections was measured for each sample.

### 2.1. Uncertainty Evaluation Procedure

To verify the quality of the measurements, an uncertainty evaluation was carried out. The measurements were carried out inside a metrological laboratory with a 20 °C ± 0.5 °C controlled temperature. The thermal deformation of the samples was neglected. The surface topography measurements were carried out using a Taylor–Hobson FORM TALYSURF Series 2 scan profilometer (Taylor Hobson, Leicester, United Kingdom). The raw surface acquisitions were post-processed with the dedicated image metrology software TalyMap (Taylor Hobson, Leicester, United Kingdom). The calibration of the scanning profilometer was performed with a calibrated roughness artifact (nominal value: *Ra* = 810 nm). According to Reference [54], it is possible to establish uncertainties for surface roughness *Sa* measurements following ISO 15530-3 [55]. 

The uncertainties for surface roughness measurements when using the induction scanning profilometer *U*_PROF_ was calculated according to the following equation:

(2) $$U_{\mathrm{PROF}}=\sqrt{{u^{2}}_{\mathrm{cal}}+{u^{2}}_{p\;}+{u^{2}}_{\mathrm{res},\;\mathrm{PROF}\;}}$$

where *u*_cal_ is the standard calibration uncertainty of the roughness standard; *u*_p_ is the standard uncertainty related to the measurement procedure and is calculated as a standard deviation of ten repeated measurements on the calibrated standard; and *u*_res,PROF_ is the resolution standard uncertainty related to the declared 3 nm vertical resolution of the scanning profilometer for measuring range 0.2 mm.

The expanded uncertainty of the surface roughness *Sa* measurement was calculated as follows:

(3) $$U_{95,\mathrm{Sa}}=k\;\sqrt{{U^{2}}_{\mathrm{PROF}}+{U^{2}}_{Sa,\mathrm{EDM}}}$$

where *k* is the coverage factor, equal to 2 for a 95% confidence interval, *u_S_*_*a*,EDM_ is calculated using the standard deviation of repeated *Sa* measurements on the electrical discharge machining sample. Table 1 presents the uncertainty budget.

The measurements of the thickness of the white layer were carried out using a Nikon Eclipse LV 150 optical microscope, coupled to an NIS-Elements BR 3.0 image analyzer (Nikon). The uncertainty was calculated using the above method. The calibrated slide with 10 µm division was selected as the calibrated artifact.

The uncertainties for the thickness of the white layer when using optical microscope *U*_OM_ was calculated according to the following equation:

(4) $$U_{\mathrm{OM}}=\sqrt{{u^{2}}_{\mathrm{cal}}+{u^{2}}_{p\;}+{u^{2}}_{\mathrm{res},\;\mathrm{OM}}}$$

where *u*_cal_ is the standard calibration uncertainty of the calibration slide, *u*_p_ is the standard uncertainty related to the measurement procedure and is calculated as the standard deviation of ten repeated measurements on the calibrated slide; and *u*_res,OM_ is the resolution standard uncertainty related to the objective magnification (50x). The expanded uncertainty of the thickness of the white layer measurement was calculated as follows:

(5) $$U_{95,\mathrm{WL}}=k\;U_{\mathrm{OM}}\;$$

where *k* is the coverage factor, equal to 2 for a 95% confidence interval. Table 2 presents the uncertainty budget.

The material removal rate (*MRR*) was calculated based on the volume of material removed from the workpiece divided by the machining time:

(6) $$\mathrm{MRR}=\frac{m_{1}-m_{2}}{\rho \;\Delta t}\;\left[\frac{mm^{3}}{min}\right]$$

where $m_{1}$ is the sample weight before processing, $m_{2}$ is the sample weight after processing, ρ is specific material density, Δt is a time of manufacturing.

Each sample was weighed before manufacturing on a precision electronic balance (Radwag, Radom, Poland). The samples after the EDM process were cleansed with the compressed air and then weighed again. The measurement uncertainties for the weight measurements were calculated according to the following equation:

(7) $$U_{B}=\sqrt{{u^{2}}_{m1}+{u^{2}}_{\mathrm{res}}+{u^{2}}_{i\;}+{u^{2}}_{\mathrm{ie}\;}}$$

where *u_m_*_1_ is the standard uncertainty related to the measurement procedure and is calculated as the standard deviation of the ten repeated measurements of the sample, *u*_res,_ is the resolution uncertainty related to the declared resolution of the balance with a readability 0.01 mg for the measuring samples of a maximum capacity 50 g, *u*_i_ is the uncertainty related to a balance indication error, and *u*_ie_ is the uncertainty on a determining indication error.

The expanded uncertainty of the weight measurement was calculated as follows:

(8) $$U_{95,W}=k\;U_{B}\;$$

where *k* is the coverage factor, equal to 2 for a 95% confidence interval. Table 3 reports the uncertainty budget.

### 2.2. Analyses of Current and Voltage Waveforms.

The complexity of the physical phenomena of the EDM process and its conditions caused considerable difficulties in describing and identifying the impact of individual machining parameters on the surface roughness, integrity, and the MRR. In the first stage of the conducted research, the measurement circuit was developed to determine the current–voltage characteristics of the generator machines. A primary test was conducted to investigate a range of stability discharges for different values of the discharge current, pulse time, and time interval. The measurement of the current and voltage waveforms in the EDM process conditions was done using a National Instruments NI5133 oscilloscope card (National Instruments, Austin, TX, USA). An application was developed in the LabView environment, which enabled the control of the work of the oscilloscope card. The current measurement was done using the indirect method as the voltage drop on the non-inductive current sensor. The maximum value of the voltage drop for the set current values did not exceed 3V, so the signal was fed directly to the oscilloscope card. The measurement of the voltage during the electric discharge was done with the Tektronix probe (Tektronix UK Ltd., Berkshire, UK). The sampling rate was 100 MS/s, 2-Channel registration. Analyses of the obtained data were performed in DIAdem (National Instruments).

**Figure 2:**
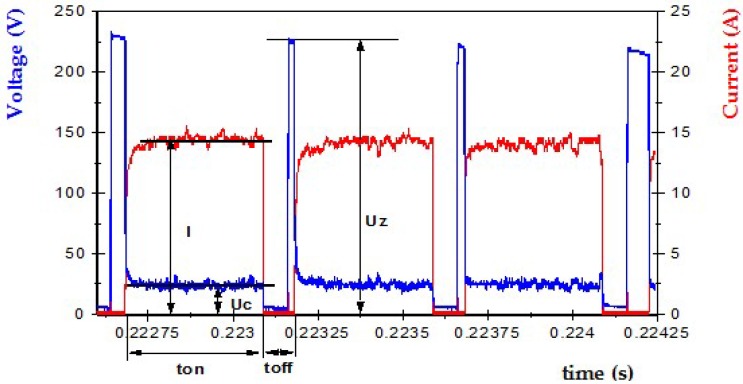
The recorded current and voltage waveforms.

Exemplary current and voltage waveforms registered for the investigated EDM are shown in Figure 2. The workpiece was machined at the moment when the supply voltage *U*z dropped to the discharge voltage *U*c, with the increase of the discharge current *I*, during the pulse *t*_on_. Then, during the *t*_off_ interval, the conditions in the gap stabilized, and the process was cyclically repeated.

The following parameters can characterize the voltage–current waveforms (Figure 2):

- *I* = the height of the peak current during discharging,
- *U*z = open circuit voltage, this is the system voltage when the EDM circuit is in the open state, and the energy has been built up for discharge,
- *U*c = discharge voltage,
- *t*_on_ = pulse time, the time required for the current to rise and fall during discharging,
- *t*_off_ = time interval, this is the time from the end of one pulse to the beginning of the next pulse with the current.

**Figure 3:**
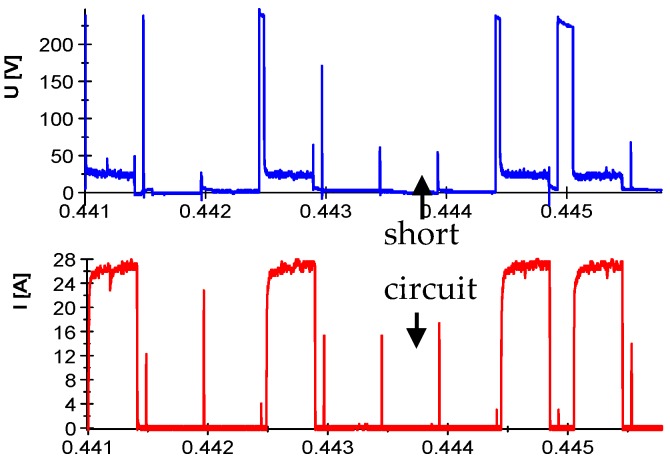
The recorded current and voltage waveforms.

Analysis of the obtained voltage and current waveforms showed that at the highest adjustable currents, pulse duration, and minimum values of the break times, in most cases, arc discharges or short circuits occurred. For short time intervals, *t*_off_, the plasma channel may not be completely deionized, which increases the probability of another discharge being in the same place. Furthermore, the ineffective removal of the products of erosion from the gap causes a reduction in the dielectric resistance and destabilization of the conditions. There is a high probability of a short circuit. The machine's control system resists the phenomena described above by increasing the gap (temporarily raising the electrode), whilst at the same time extending the break time (Figure 3). 

**Figure 4:**
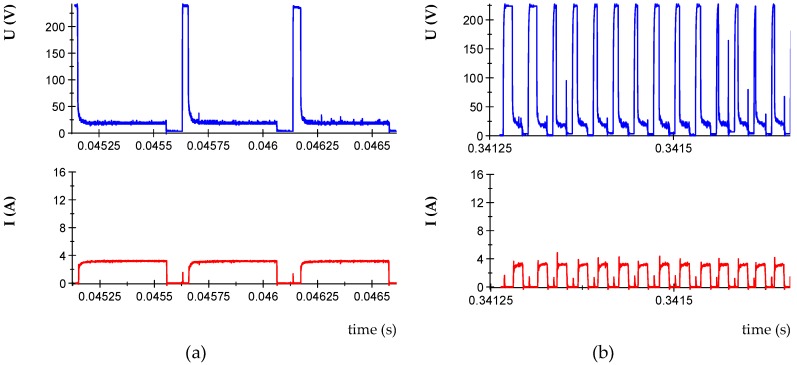
The recorded voltage and current waveforms for the following parameters: (**a**) *U*_c_ = 25 V, *U_z_ =* 230 V, *I =* 3 A, *t*_on_ = 400 µs, *t*_off_ = 100 µs; (**b**) *U*_c_ = 25 V, *U*_z_ = 230 V, *I* = 3 A, *t*_on_ = 13 μs, *t*_off_ = 13 μs.

The proper operation of the generator control system ensures the energy repeatability of discharges. Therefore, the presented disturbances will not have a significant impact on the quality of the treated surfaces. Nevertheless, unfavorably selected ranges of the set parameters will significantly affect the efficiency of the process [56]. The following are examples of stable *U*(*t*), *I*(*t*) waveforms, which enabled the selection of the range of variability of parameters used in the experimental research (Figure 4). 

Analysis of the recorded voltage and current waveforms enabled the selection of stable parameters in the EDM process for roughing, semi-finishing, and finishing machining. Experimental studies were conducted using the orthogonal full factorial design DOE (design of experiments) methodology: Orthogonal full factorial design. Preliminary experiments were conducted to obtain the stable discharges in all ranges of the design matrix. After analysis of the results, the following machining conditions were selected: discharge current in the range *I* = 3–14 A, pulse time in the range *t*_on_ = 10–400 µs, and time interval *t*_off_ = 10–150 μs, with the following constants :open voltage *U*_0_ = 225 V, discharge voltage *U*c = 25 V. Table 4 shows the levels of machining parameters carried out in the experimental design. 

## 3. Results and Discussion

### 3.1. Analysis of Surface Integrity

**Figure 5:**
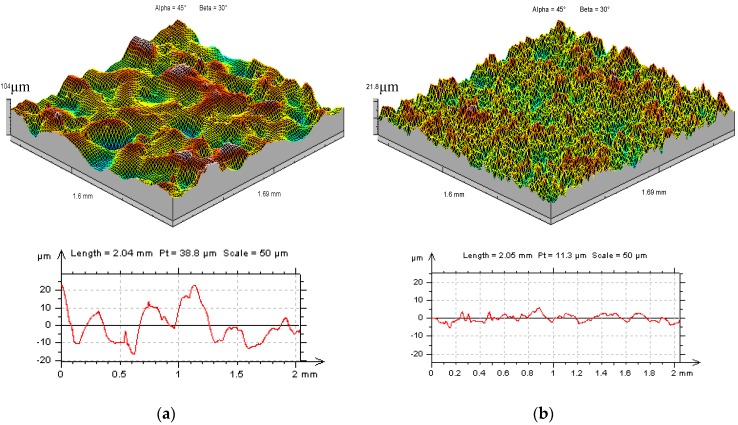
The surface texture of the tool steel 55NiCrMoV7 after electrical discharge machining (EDM): (**a**) *U_c_* = 25 V, *I* = 8.5 A, *t*_on_ = 400 µs, *t*_off_ = 150 µs; (**b**) *U_c_* = 25 V, *I* = 3 A, *t*_on_ = 400 µs, *t*_off_ = 80 µs.

The primary factor that affected the properties of the machined parts was the surface texture. Experimental investigations showed that parameters such as surface roughness (*Sa*), directly depended on the applied machining parameters. Surface topography after EDM was the result of the overlapping of craters from single discharges and it had an isotropic structure (Figure 5). 

**Figure 6:**
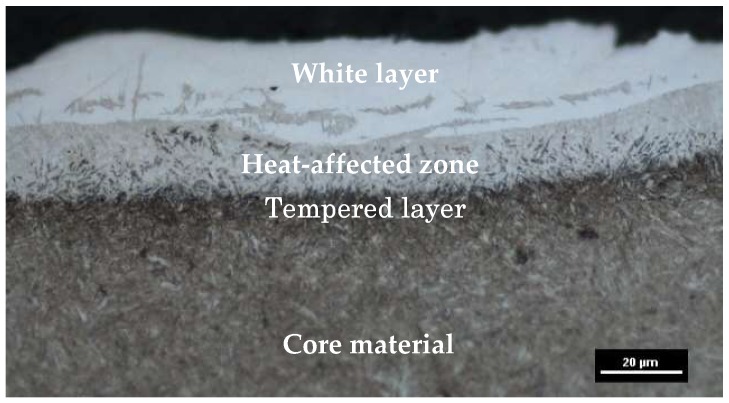
The metallographic structure of tool steel 55NiCrMoV7 after (EDM).

Owing to rapid local thermal processes during electrical discharge machining, phase changes occurred on the surface layer of the workpiece. The analysis of images of the metallographic structure of tool steel 55NiCrMoV7 showed the occurrence of three characteristic sublayers for the whole range of the investigated machining parameters (Figure 6).

**Figure 7:**
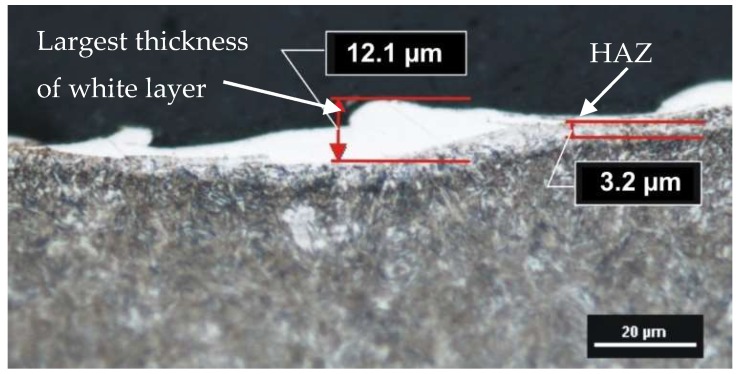
The metallographic structure of tool steel 55NiCrMoV7 after EMD: *U*c = 25 V, *I* = 14 A, *t*_on_ = 13 µs, *t*_off_ = 10 µs.

An external molten layer (commonly referred to as a white layer), was formed by melting and rapidly solidifying a thin layer of metal not removed from the surface of the crater during an electric discharge. The white layer in its structure may have chemical decompositions from both the core material and the working electrode. The heat-affected zone (HAZ), which is located directly under the melted layer (visible as a light structure), was characterized by an increased hardness around the core material. The last observed layer was the tempered layer, which was visible as a dark streak immediately below the heat-affected zone layer. The thickness of each observed layer depended on the investigated EDM parameters. The white layer, unlike the heat-affected zone and the tempered layer, was characterized by local discontinuities and thickness changes (Figure 7). The industrial application of the results of the conducted experiments requires information on the largest thickness of the white layer. This information allows for the correct selection of machining allowances for semi-finishing and finishing manufacturing.

**Figure 8:**
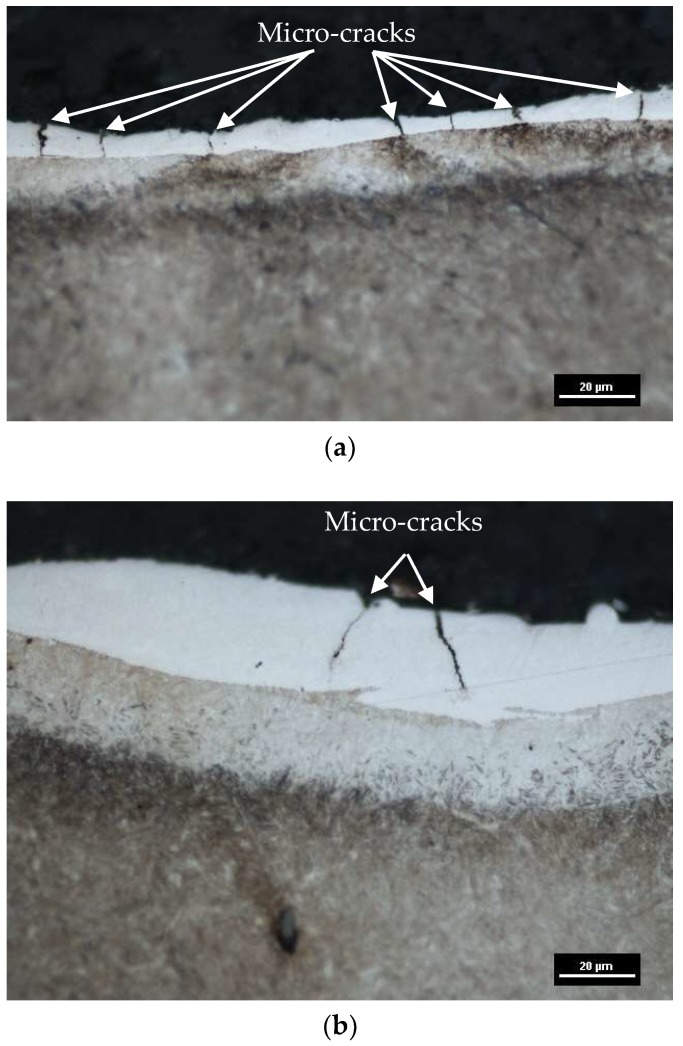
The metallographic structure of tool steel 55NiCrMoV7 after EMD: (**a**) *Uc* = 25 V, *I* = 3 A, *t*_on_ = 206 µs, *t*_off_ = 80 µs; (**b**) *Uc* = 25 V, *I* = 14 A, *t*_on_ = 400 µs, *t*_off_ = 80 µs.

**Figure 9:**
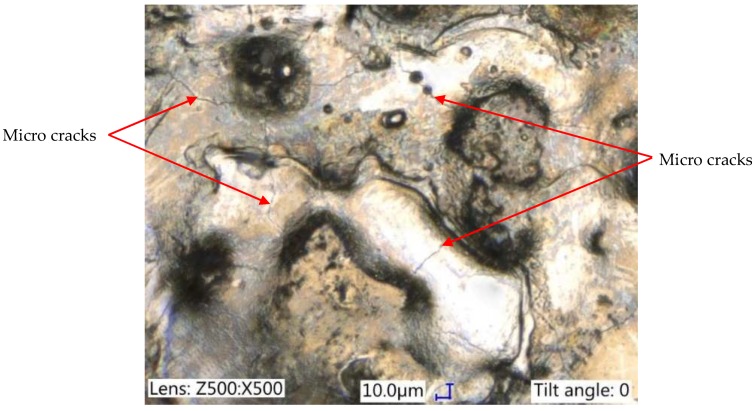
The structure of the surface after EMD: *U*c = 25 V, *I* = 14 A, *t*_on_ = 400 µs, *t*_off_ = 150 µs.

Electrical discharges caused the local melting and evaporation of material. The thermal processes of removing material and the rapid re-solidification of the molten metal which was not removed from the discharge crater generated thermal stress. Exceeding the maximum tensile strength of the material caused the generation of micro-cracks. Micro-cracks are an undesirable effect, resulting in reduced fatigue resistance and corrosion resistance. In most cases, micro-cracks propagate to the end of the white layer (Figure 8). In rare cases, the propagation of a crack has been observed to penetrate the core of the material. Micro-cracks can be observed directly on the machining surface (Figure 9). 

### 3.2. Response Surface Methodology

Experimental investigation of the influence of the EDM parameters on the surface roughness (*Sa*), the thickness of the white layer, and the MRR was carried out using response surface methodology. In RSM, the dependence between the desired response and the independent variables can be represented by the following:

*Y* = *f* (*I*,*t*_on_, *t*_off_) ± *ε* (9)

where *Y* is the response; *f* is the response function; *ε* is the experimental error. *I* the discharge current, *t*_on_ (μs) the pulse time, and *t*_off_ (μs) the time interval are independent parameters. In the study, the polynomial regression model was chosen to fit the response function to the experimental results. 

The experimental investigation was carried out based on the full factor orthogonal experiment design: three-level three-parameter. The study of the influence of the input factors on three equidistant levels of variation allows for the determination of regression equations with a high degree of correlation and a small spread of values. According to the full factor orthogonal design plan, twenty-eight samples, with one additional replication in the center point, were manufactured and measured. Based on the experimental data, an empirical model of the influence of the discharge current *I*, pulse time *t*_on_, and time interval *t*_off_ was built. The results of the experimental studies are presented in Table 5. The surface roughness (*Sa*) was in the range of 1.88 µm to 12.7 µm. The maximal thickness of the white layer was in the range of 5.5 µm to 33.5 µm. The material removal rate was in the range 0.1 mm^3^/min to 29.19 mm^3^/min. The obtained value of roughness (*Sa*), the maximal thickness of the white layer, and the *MRR* corresponded to the finishing and roughing machining. 

Analysis of variance (ANOVA) was used to check the significance of each independent variable in the response function. The ANOVA test was conducted at a 5% significant level. The *F*-value corresponded to a continuous probability distribution. If this probability (Prob > *f*) value for each factor was less than 0.05, this indicated that the model factor was significant (i.e., at a 95% confidence level). Values of Prob > *f* higher than 0.05 indicated that a model factor was non-significant. 

The ANOVA results for the *Sa*, white layer thickness, and the MRR are shown in Table 6, Table 7 and Table 8, respectively. Table 6 shows the ANOVA results for surface roughness (*Sa*). The calculated contribution indicated that the discharge current had the most influence on the surface roughness (*Sa*) (57.6%). Second, the affecting variable was pulse time (15.7%) and the interaction of the discharge current with pulse time (14.5%). Other variables and their interactions had a significant influence on the surface roughness (*Sa*), but each of the contributions did not exceed 5%. The ANOVA results presented in Table 7 indicated that the most significant influence on the maximal thickness of the white layer was the discharge current (47.5%), followed by the pulse time (27.8%) and the squared pulse time (9.2%). The contribution of other variables on the *Wl* was significant but less important. Table 8 presents the ANOVA results for the MRR. The calculated contributions indicated that the discharge current (55.9%) had the most influence on the MRR, followed by the interaction of the discharge current with the pulse time (12.7%) and also the pulse time (11.6%). Other variables and their interactions were significant, but their contributions were smaller and contained in the range (1.6%–5.6%). From the presented ANOVA Table 6, Table 7 and Table 8, calculated Fisher coefficients for the models *Sa*, WL, and MRR were 150.95, 141.26, and 208.65, respectively. The results implied that all the developed models were significant at a 95% confidence level.

Regression analysis with a backward elimination process was performed. For each equation, we calculated the coefficient of determination, *R-squared*, and the adjusted coefficient of determination, *R-Adj*. The coefficients represented the percentage of variance explained by the model. When the value of the *R-sqr* and *R-Adj* approaches unity, a more accurate fit of the regression equation to the research results would be obtained.

After eliminating the non-significant factors in the response equations for the surface roughness (*Sa*), maximal white layer thickness (WL), and MRR, this was described by the following polynomial function:

*Sa* = 0.38 + 0.54 *I* − 0.027 *I*^2^ − 0.0004 *t*_on_ + 0.00002 *t*_on_^2^ + 0.00006 *I t*_on_ − 0.000007 *I t*_on_^2^*+ 0.0003I*^2^*t*_on_ (10)

WL = 1.422 + 1.697 *I* − 0.075 *I*^2^ − 0.101 *t*_on_*+* 0.0003 *t*_on_^2^ − 0.002 *t*_off_*+* 0,035 *I t*_on_ − 0.0001 *I t*_on_^2^ − 0.0014 *I*^2^*t*_on_ + 0.000005 *I*^2^*t*_on_^2^ + 0.00027 *t*_on_*t*_off_ − 0.000001 *t*_on_*^2^ t*_off_ (11)

MRR = −1.2087 + 0.342 *I* + 0.02967 *I*^2^ − 0.00817 *t*_on_ + 0.00004 *t*_on_^2^ + 0.02287 *t*_off_ + 0.00096 *I t*_on_ − 0.00001 *I t*_on_^2^ + 0.00057 *I*^2^*t*_on_ − 0.00668 *I t*_off_ (12)

**Figure 10:**
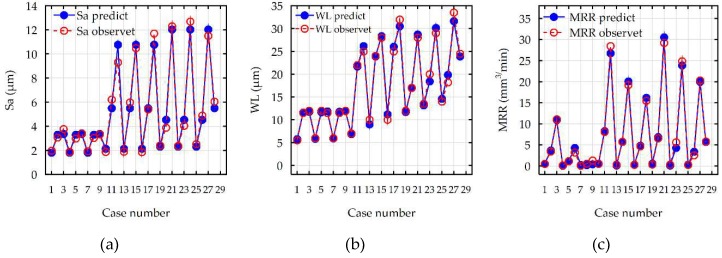
The comparison between the results of experimental studies to the values calculated based on the developed models for (**a**) surface roughness (*Sa*); (**b**) maximal thickness of white layer (WL); (**c**) material removal rate (MRR).

Analyses of the results of the MRR showed that the values of the *R*-squared for surface roughness (*Sa*), the maximal thickness of the white layer, and the MRR were over 98%, 99%, and 99%, respectively. This result indicated that the regression models provided an excellent explanation of the relationship between the independent variables and the response *Sa*, WL, and MRR. Differences between the *R*-squared and the *R-*adjustable were smaller than 0.2, which indicated that the established model was adequate in representing the process. The developed models can be used to predict the values of surface roughness (*Sa*), the maximal value of white layer thickness (WL), and the material removal rate (MRR). Comparisons between the results of experimental studies and the values calculated based on the developed models for *Sa*, WL, and MRR are shown in Figure 10. The results indicated that the predicted values were very close to the experimental data. 

**Figure 11:**
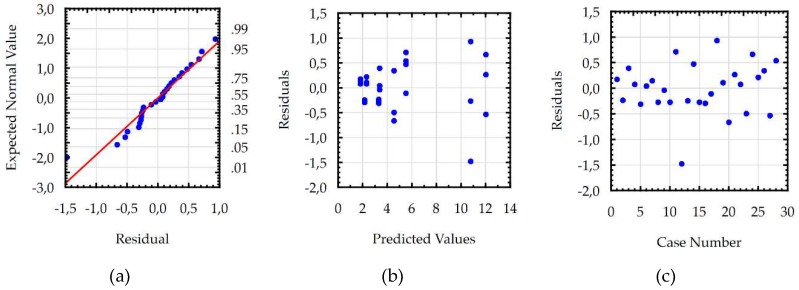
The plots to check the model for surface roughness (*Sa*): (**a**) the normal plot of residuals; (**b**) the residuals versus the predicted values; and (**c**) the residuals versus the case values.

**Figure 12:**
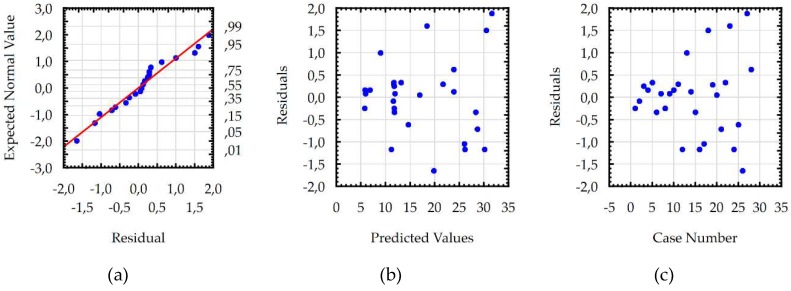
The plots to check the model for maximal white layer thickness: (**a**) the normal plot of residuals; (**b**) the residuals versus the predicted values; and (**c**) the residuals versus the case values.

**Figure 13:**
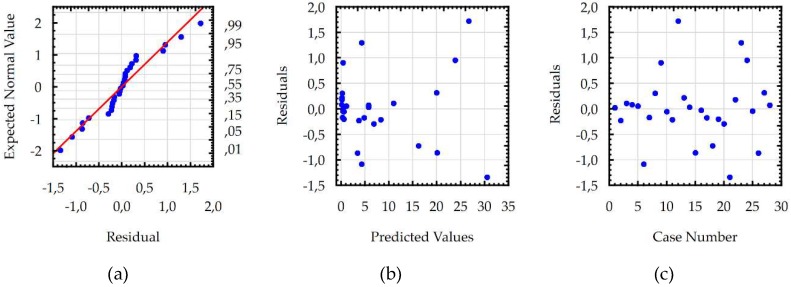
The plots to check the model MRR: (**a**) the normal plot of residuals; (**b**) the residuals versus the predicted values; and (**c**) the residuals versus the case values.

Developed models for the *Sa,* WL, and MRR were checked using additional statistical tests, which confirmed the basic assumptions using the ANOVA. Residuals had a normal distribution, constant variance, and were independent of an order of data. The assumption of constant variance was checked by plotting the residuals versus the predicted values. The normality assumption and independence of residuals were checked by plotting the expected normal value versus the residuals and the residuals versus the order of data, respectively. Analysis of the residual normal probability plots (Figure 11a, Figure 12a and Figure 13a) showed that the residuals had normal distributions. Plots of the residuals versus the predicted values (Figure 11b, Figure 12b and Figure 13b); and the residuals versus the case number values (Figure 11c, Figure 12c and Figure 13c) showed that the residuals had a stochastic nature. The analysis of the plotted residuals versus the case values indicated that the error terms were independent of one another. The analyses of the residuals confirmed that the developed models were adequate.

**Figure 14:**
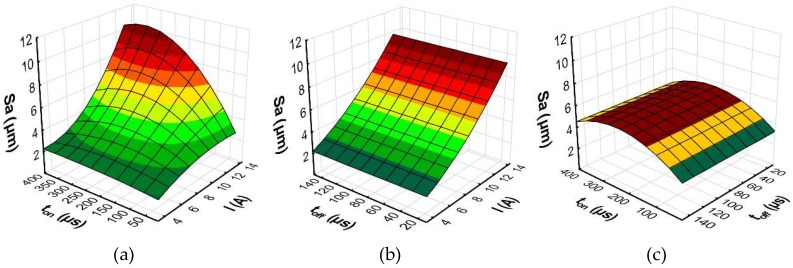
The estimated response surface plot for roughness (*Sa*): (**a**) constant *t*_off_ = 80 µs; (**b**) constant *t*_on_ = 206 µs; and (**c**) constant *I* = 8.5 A.

**Figure 15:**
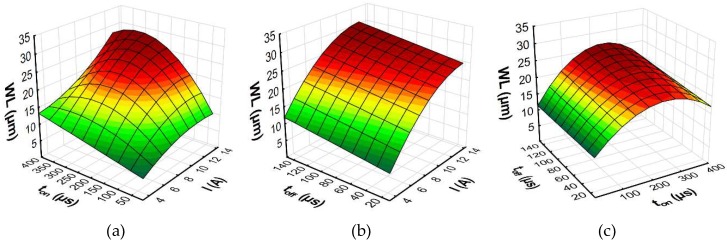
The estimated response surface plot for the WL: (**a**) constant *t*_off_ = 80 µs; (**b**) constant *t*_on_ = 206 µs; and (**c**) constant *I* = 8.5 A.

**Figure 16:**
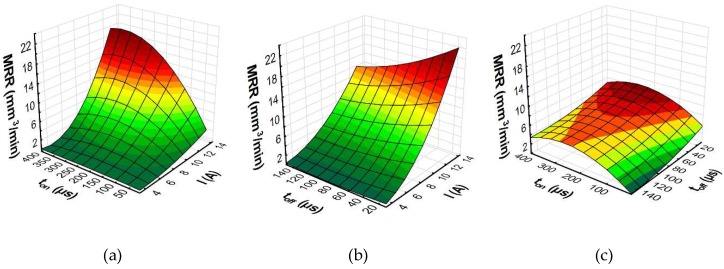
The estimated response surface plot for the MRR: (**a**) constant *t*_off_ = 80 µs; (**b**) constant *t*_on_ = 206 µs; and(**c**) constant *I* = 8.5 A.

To better understand the influence of the EDM parameters on surface roughness (*Sa*), the maximal thickness of white layer, and the material removal rate, the response surface plots were estimated. Based on the regression models (Equations (10)–(12)), the influence of the discharge current *I,* pulse time *t*_on_, and time interval *t*_off_ on the *Sa*, WL, and MRR is shown in Figure 14, Figure 15 and Figure 16, respectively. 

The results of the experimental studies indicated that the main parameters that influenced surface roughness (*Sa*), were the discharge current and the pulse time (Figure 14). Time interval, in the case of stability discharges, does not have a significant impact on the surface texture properties. The surface roughness (*Sa*) increases with the growth of the discharge current and the pulse time. These two parameters, with constant voltage, determine the amount of energy of the electrical discharge. At the lowest value of the discharge current, the changing of the pulse time does not generate a crater with greater depth. The surface roughness (*Sa*) does not change significantly. With the increase of the discharge current, the amount of energy delivered to the workpiece causes the melting and evaporation of a higher volume of material, which generates a crater with a larger depth. The presented dependence also has effects on the material removal rate. The MRR, similar to surface roughness, is influenced by the volume of material which is removed in single discharges, and it mainly depends on the discharge current (Figure 16). The time interval is responsible for the stabilization of the conditions in the gap after discharges. In a stable EDM process, increases of the time interval result in the decrease of the material removal rate. Discharge energy devoted to the heat flux, the mechanism of growing the plasma channel, and the removal process of the material implies the thickness of the white layer. Figure 15 shows the estimated response surface plot for the white layer thickness in relation to the EDM parameters. The increase in the pulse time and current resulted in an increase in the amount of melted and evaporated material from the discharge zone. However, more material which was melted in the single crater was not removed from the surface of the workpiece and it re-solidified on the core. 

In the industrial application of the developed models, splitting the electrical discharge machining into several steps should be considered. In the first stage, the material will be removed from the workpiece using the highest discharge energy (roughing technology). In the roughing step, the applied parameters should ensure the maximum removal rate. In the next step, the parameters of the process should be changed to achieve proper surface layer properties and a low roughness for the machined surface of the manufactured parts. The EDM process will be conducted with the semi-finishing and finishing step, with respectively lower discharge energies. In the last step—the finishing treatment—it is vital to obtain the appropriate surface roughness and thickness of the white layer. However, the finishing can take more time than the roughing. The result is that a significant increase in the cost of production may be observed. In the finishing machining, a combination of minimum surface roughness with the minimum value of white layer thickness, and with a possibly maximum *MRR* is desirable. In the case of EDM, the simultaneous achievement of these three goals is conflicting. For that reason, in considering the properties of the EDM optimization, it should be based on the desirability technique. This method uses the Derringer's [57] desirability function, which in the case of the same importance of each response can be described by the equation:

(13) $$D={\left(d_{1}\times d_{2}\times .....\times d_{n}\right)}^{1/n}={\left(\prod_{i=1}^{n}d_{i}\right)}^{\frac{1}{n}}$$

where *n* is the number of responses in the measure.

The desired function is established for each investigated response, *d_i_*(${\hat{y}}_{i}$), and it has a range from zero to one (one being the most desirable). Different desirable functions can be built, depending on the adopted optimization criteria which determine the desirable value, maximal (upper-*U_i_*) or minimal (lower-*L*_i_). If the response for the investigated parameter should be minimized, then *d_i_*(${\hat{y}}_{i}$) can be calculated according to the following equation:

(14) $$d_{i}({\hat{y}}_{i})=\{\begin{array}{ll} 1 & {\hat{y}}_{i}<L_{i}\\ {\left(\frac{U_{i}-{\hat{y}}_{i}}{U_{i}-L_{i}}\right)}^{t}, & L_{i}\leq {\hat{y}}_{i}\leq U_{i}\\ 0 & {\hat{y}}_{i}>U_{i} \end{array}$$

If the desirable function should be maximized, then it can be expressed by the following equation:

(15) $$d_{i}({\hat{y}}_{i})=\{\begin{array}{ll} 0 & {\hat{y}}_{i}<L_{i}\\ {\left(\frac{{\hat{y}}_{i}-L_{i}}{U_{i}-L_{i}}\right)}^{s}, & L_{i}\leq {\hat{y}}_{i}\leq U_{i}\\ 1 & {\hat{y}}_{i}>U_{i} \end{array}$$

**Figure 17:**
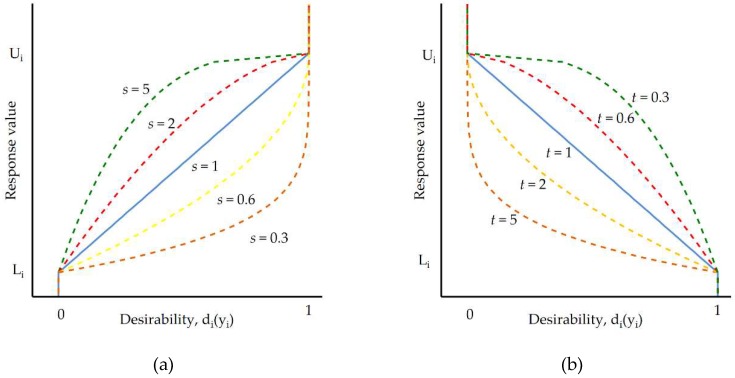
The desirability functions for the target value: (**a**) maximized; (**b**) minimized criteria.

Calculations of the desirable function consider the extent to which the estimated values (${\hat{y}}_{i}$) are close to the minimum or maximum. Figure 17 presents a graphical interpretation of the desirability functions with the “importance” levels *s* and *t*. When considering the case when the “importance” *t* (for minimum) and *s* (for maximum) is large, the desirability is low unless the response moves close to the target. For low-value parameters, *t* and *s* desirability has a high value for a wide range of responses. It means that it is possible to achieve satisfactory desirability not only in the target value (minimum *L_i_* or maximum *U_i_*).

The multi-response optimization was divided into three cases. In the first case, we examined the optimal parameters of the finishing EDM. For this task, the goal was defined as minimizing the surface roughness and white layer thickness, whilst maximizing the maximum material removal rate. The second case was to find the optimal parameters for semi-finishing EDM. In this task, we aimed to achieve an average value of the MRR (about 14.5 mm^3^/min), with the possibility of minimizing the surface roughness and white layer thickness. In the last case, the optimal parameters for roughing EDM were considered. In this task, the goal of optimization was to achieve the maximum MRR possible, with the possibility of minimizing the surface roughness and white layer thickness. These three cases of the optimization of electrical discharge machining were carried out using the desirability function, based on the regression Equations (10)–(12). It should be maintained that the success of the optimization with the desirability function mainly depends on the quality of the regressions models. In this study, each established model had a coefficient of determination, *R-squared,* that was over 98%, and the differences between the *R*-squared and the *R*-adjustable were smaller than 0.2, which indicated that the models were adequate in representing the process. For each EDM parameter (discharge current, pulse time, time interval) there was a simultaneous analysis of every combination of the factors for each of the nine responses (Figure 14, Figure 15 and Figure 16). A multi-response optimization procedure was performed for the global desirability function. The ranges for the constraints and factors for optimization are shown in Table 9.

**Figure 18:**
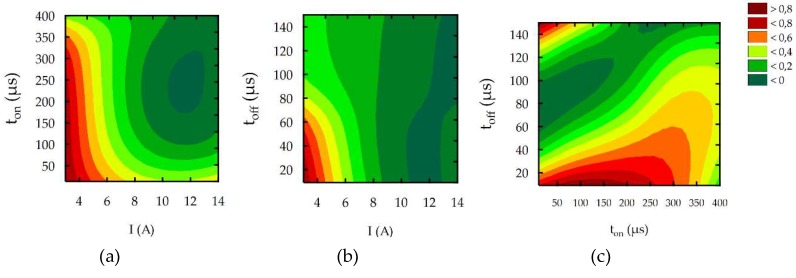
The contour plots of the desirability function for the finishing EDM optimization.

**Figure 19:**
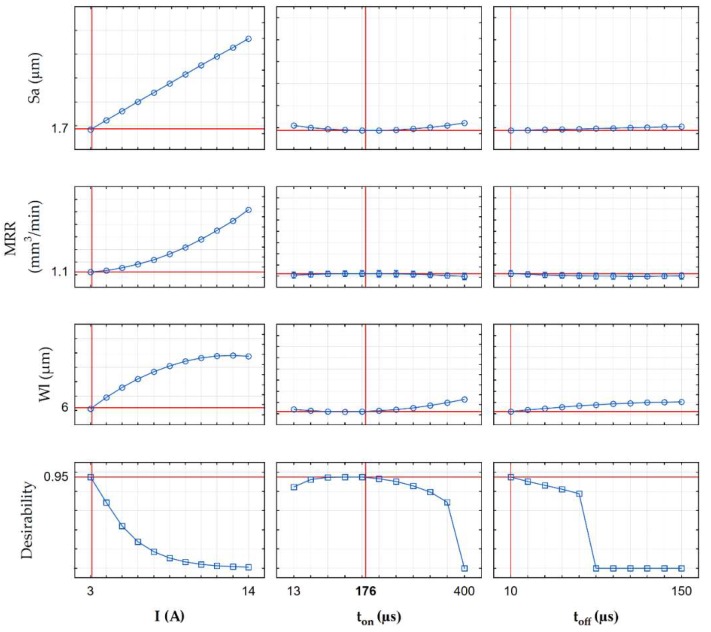
The example of profiles for the predicted values and desirability for the finishing EDM.

**Figure 20:**
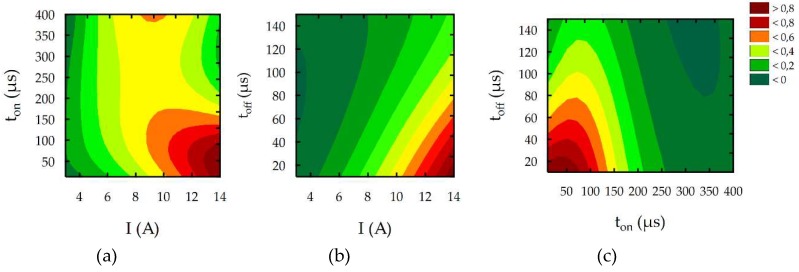
The contour plots of the desirability function for semi-finishing EDM optimization.

**Figure 21:**
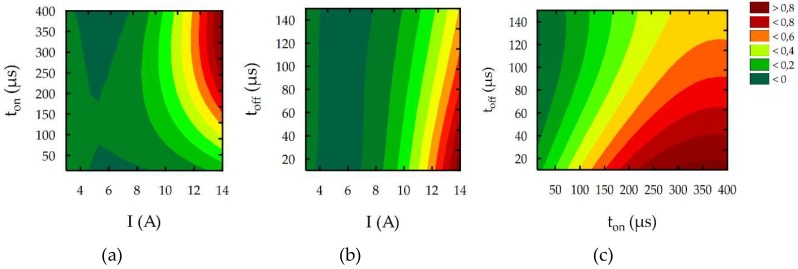
The contour plots of the desirability function for roughing EDM optimization.

The results of the multi-response optimization procedure of the global desirability function for the finishing, semi-finishing, and roughing operations are shown in the contour plots in Figure 18, Figure 19, Figure 20 and Figure 21, and in Table 10. The desirable function in the first case (finishing EDM) reached 0.95 (Figure 19). The semi-finishing and roughing operations reached 0.98 and 0.99, respectively. The optimal EDM parameters for finishing electrical discharge machining were a discharge current *I* = 3 A, pulse time *t*_on_ = 176 µs, and pulse interval *t*_off_ = 10 µs. The predicted surface roughness (1.7 µm) and the white layer thickness (6 µm) after optimization were close to the results obtained in the experimental studies for sample number four. Nevertheless, the material removal rate grew almost seven times and reached an MRR = 1.1 mm^3^/min. The increase of the MRR was achieved, along with the minimization of the surface roughness and the white layer thickness, which has a significant effect on productivity.

Figure 20 and Table 10 present the results of the optimization for semi-finishing EDM. Values of the optimal EDM parameters were as follows: discharge current *I* = 14 A, pulse time *t*_on_ = 52 µs, and pulse interval *t*_off_ = 24 µs. In this case, if the material removal rate reached 14.5 mm^3^/min (i.e., the average value of the MRR from experimental studies), the optimized surface roughness and the white layer thickness were as follows: *Sa* = 5.2 µm and WL = 15 µm. Figure 21 and Table 10 present the results of the optimization for roughing EDM. Values of the optimal EDM parameters as follows: *I* = 14 A, *t*_on_ = 361 µs, and *t*_off_ = 24 µs. The optimized material removal rate, surface roughness, and white layer thickness were as follows: MRR = 29.2 mm^3^/min, *Sa* = 12.1 µm, WL = 28.8 µm. 

In the last stage of the experimental investigations for the optimal EDM parameters, a confirmation test was conducted on the EDM machine Charmilles Form 2LC ZNC (Bern, Switzerland). Table 10 presents the results of the validations of multi-response optimizations. The maximal errors between the predicted and the obtained values were 6%, which could be considered a very good result. The calculated measurement uncertainty (at 95% confidence) of the *Sa* and MRR is much lower than the measurement uncertainty of the thickness of the white layer. Moreover, prediction errors are in the range of maximal measurement uncertainty.

## 4. Conclusions

In this study, the EDM of tool steel 55NiCrMoV7 was analyzed and optimized for three cases: roughing, semi-finishing, and finishing machining. Based on the theoretical analyses and experimental research, the following conclusions were obtained:

1. Experimental research on the influence of discharge current, pulse time, and pulse interval on the surface roughness (*Sa*), white layer thickness, and the MRR showed that the discharge current had the main effect on *Sa*, WL, and the MRR. With an increase in the discharge current and pulse time, the amount of energy delivered to the workpiece caused the melting and evaporation of a higher volume of material, which generated craters with a larger depth and diameter. However, more material which melted in the single crater was not removed from the surface of the workpiece and it re-solidified on the core. The time interval between pulses did not significantly affect the change in surface integrity and the MRR, but it played an important role in the stability of the process.
2. The desirability function was used in the multi-response optimization of three functions: *Sa*, WL, and MRR. For the three cases of EDM—finishing, semi-finishing, and roughing operations—the optimal parameters were established. The confirmation tests for the established optimal parameters showed that the maximal errors between the predicted and the obtained values did not exceed 6%, which could be considered as a very good result.
3. The developed regression equations could be used in electrical discharge machining as a guideline for the selection of EDM parameters.

## Figures and Tables

**Table 1:** The uncertainty contributions for the *Sa* roughness measurements on the scanning profilometer.

Uncertainty Contributions (nm)					
*u*_cal_	*u*_p_	*u*_res,PROF_	*U*_PROF_	*U_Sa, EDM_*	*U*_95,Sa_
20	2	3	20.5	5	42

**Table 2:** The uncertainty contributions for the thickness of the white layer measurements on microscope.

Uncertainty Contributions (μm)				
*u*_cal_	*u*_p_	*u*_res,OM_	*U*_OM_	*U*_95,WL_
0.060	0.048	0.312	0.321	0.6

**Table 3:** The uncertainty contributions for the weight measurements on the precision balance.

Uncertainty Contributions (mg)					
*u_m_*_1_	*u*_res_	*u*_i_	*u*_ie_	*U*_B_	*U*_95,W_
0.02	0.0029	0.0058	0.01	0.023	0.046

**Table 4:** The levels of machining parameters carried out in the experimental design.

EDM Parameters	Level 1	Level 2	Level 3
discharge current *I* (A)	3	8.5	14
pulse time *t*_on_ (μs)	13	206	400
time interval *t*_off_ (μs)	9	80	150

**Table 5:** The design of the experimental matrix.

Exp. no.	EDM Parameters			Observed Values		
	Discharge Current *I* (A)	Pulse Time *t*_on_ (μs)	Time Interval *t*_off_ (μs)	Surface Roughness *Sa* (μm)	Maximal Thickness of the White Layer (μm)	MRR (mm^3^/min)
1	3	13	10	2.0	5.5	0.54
2	8.5	13	10	3.1	11.5	3.47
3	14	13	10	3.8	12	11.06
4	3	13	80	1.9	6	0.17
5	8.5	13	80	3.0	12	1.18
6	14	13	80	3.4	11.5	3.21
7	3	13	150	1.9	6	0.10
8	8.5	13	150	3.0	11.5	0.55
9	14	13	150	3.3	12	1.31
10	3	206	10	1.9	7	0.51
11	8.5	206	10	6.2	22	8.09
12	14	206	10	9.3	25.4	28.46
13	3	206	80	1.9	10	0.36
14	8.5	206	80	6.0	24	5.77
15	14	206	80	10.5	28	19.23
16	3	206	150	1.8	10	0.29
17	8.5	206	150	5.4	25	4.68
18	14	206	150	11.7	32	15.48
19	3	400	10	2.4	12	0.37
20	8.5	400	10	3.9	17	6.58
21	14	400	10	12.3	28	29.19
22	3	400	80	2.4	13.5	0.34
23	8.5	400	80	4.0	20	5.61
24	14	400	80	12.7	29	24.84
25	3	400	150	2.5	14	0.28
26	8.5	400	150	4.9	18.4	2.56
27	14	400	150	11.5	33.5	20.31
28	8.5	206	80	6.1	24.5	5.88

**Table 6:** The analysis of variance (ANOVA) table for the *Sa (*after elimination).

Source	Sum of Squares	Degrees of Freedom	Mean Square	*F*-Value	Prob > *f*	Contribution %
Model	344.7600	7	49.027	150.95	<0.0001	-
*I*	198.5468	1	198.5468	611.29	<0.0001	57.6
*I^2^*	5.8097	1	5.8097	17.88	0.0004	1.7
*t*_on_	54.1840	1	54.1840	166.82	<0.0001	15.7
*t*_on_ *^2^*	16.1085	1	16.1085	49.59	<0.0001	4.7
*I t*_on_	50.0208	1	50.0208	154.01	<0.0001	14.5
*I t*_on_ *^2^*	8.9235	1	8.9235	27.47	<0.0001	2.6
*I^2^ t*_on_	11.1696	1	11.1696	34.39	<0.0001	3.2
Error	6.4953	20	0.32479	-	-	-
Total SS	351.2560	27	*R-sqr =* 0.98		*R-Adj =* 0.97	

**Table 7:** The ANOVA table for the maximal thickness of the white layer (after elimination).

Source	Sum of Squares	Degrees of Freedom	Mean Square	*F*-Value	Prob > *f*	Contribution %
Model	1886.366	11	171.48	141.26	<0.0001	-
*I*	896.656	1	896.656	738.59	0.0022	47.5
*I* ^2^	16.041	1	16.041	13.21	<0.0001	0.8
*t*_on_	524.880	1	524.880	432.35	<0.0001	27.8
*t*_on_^2^	174.366	1	174.366	143.62	0.0002	9.2
*t*_off_	27.406	1	27.406	22.57	<0.0001	1.4
*I t*_on_	90.750	1	90.750	74.75	0.0004	4.8
*I t*_on_^2^	61.584	1	61.583	50.72	0.0031	3.3
*I* ^2^ *t*_on_	37.210	1	37.210	30.65	0.0071	2.0
*I* ^2^ *t*_on_^2^	44.018	1	44.017	36.25	<0.0001	2.3
*t*_on_ *t*_off_	5.603	1	5.603	4.61	<0.0001	0.3
*t*_on_^2^ *t*_off_	7.860	1	7.859	6.47	0.0473	0.4
Error	19.424	16	1.2140	738.59	0.0216	-
Total SS	1905.799	27	*R*-*sqr* = 0.99		*R*-*Adj* = 0.98	

**Table 8:** The ANOVA table for the material removal rate (MRR) (after elimination).

Source	Sum of Squares	Degrees of Freedom	Mean Square	*F*-Value	Prob > *f*	Contribution %
Model	2243.49	9	247.881	208.65	<0.0001	-
*I*	1253.287	1	1253.287	1055.08	<0.0001	55.9
*I* ^2^	126.243	1	126.243	106.27	<0.0001	5.6
*t*_on_	260.655	1	260.655	219.43	<0.0001	11.6
*t*_on_^2^	56.489	1	56.489	47.55	<0.0001	2.5
*t*_off_	101.381	1	101.381	85.34	<0.0001	4.5
*I t*_on_	285.948	1	285.948	240.72	<0.0001	12.7
*I t*_on_^2^	36.030	1	36.030	30.33	<0.0001	1.6
*I* ^2^ *t*_on_	44.106	1	44.106	37.13	<0.0001	2.0
*I t*_off_	79.350	1	79.350	66.80	<0.0001	3.5
Error	21.381	18	1.188	-	-	-
Total SS	2264.87	27	*R-sqr =* 0.99		*R-Adj =* 0.99	

**Table 9:** The goals and factor range for optimization.

Factors	Goal	Lower Limit	Upper Limit	Weight	Importance		
					Finishing EDM	Semi-Finishing	Roughing
*I* (A)	In range	3	14	1	-	-	-
*t_on_* (µs)	In range	13	400	1	-	-	-
*t_off_* (µs)	In range	10	150	1	-	-	-
*Sa* (µm)	Minimize	1.85	12.7	1	*t* = 5	*t* = 3	*t* = 0.3
WL (µm)	Minimize	5.5	33.5	1	*t* = 5	*t* = 3	*t* = 0.3
MRR (mm^3^/min)	Maximize	0.01	29.19	1	*s* = 0.3	*s* = 3	*s* = 5

**Table 10:** The experimental validations of the multi-response optimizations.

Optimal EDM Parameters		Summary of Values Obtained in Optimization			
		Response	Predicted	Experimental Verification	Error%
Finishing	*I* = 3 A *t*_on_ = 176 µs *t*_off_ = 10 µs	*Sa (*µm)	1.7	1.8	6
		WL (µm)	6	6.3	5
		MRR (mm^3^/min)	1.13	1.06	6
Semi- finishing	*I* = 14 A *t*_on_ = 52 µs *t*_off_ = 24 µs	*Sa (*µm)	5.2	5.4	4
		WL (µm)	15	15.8	5
		MRR (mm^3^/min)	14.5	15	3
Roughing	*I* = 14 A *t*_on_ = 361 µs *t*_off_ = 24 µs	*Sa* (µm)	12.1	12.7	5
		WL (µm)	28.8	30.5	6
		MRR (mm^3^/min)	29.2	28.1	4
